# Properties and Applications of the β Phase Poly(vinylidene fluoride)

**DOI:** 10.3390/polym10030228

**Published:** 2018-02-26

**Authors:** Liuxia Ruan, Xiannian Yao, Yufang Chang, Lianqun Zhou, Gaowu Qin, Xianmin Zhang

**Affiliations:** 1 Key Laboratory for Anisotropy and Texture of Materials (Ministry of Education), School of Material Science and Engineering, Northeastern University, Shenyang 110819, China; ruanlx948536015@yeah.net (L.R.); 1670523@stu.neu.edu.cn (X.Y.); qingw@smm.neu.edu.cn (G.Q.); 2 Computer Teaching and Researching Section, Shenyang Conservatory of Music, Shenyang 110818, China; changyf537@126.com; 3 Suzhou Institute of Biomedical, Engineering and Technology, Chinese Academy of Sciences, Suzhou 215163, China; zhoulq@sibet.ac.cn

**Keywords:** β phase PVDF, electroactive properties, nanofilms, sensor and actuator, spin valve devices

## Abstract

Poly(vinylidene fluoride), PVDF, as one of important polymeric materials with extensively scientific interests and technological applications, shows five crystalline polymorphs with α, β, γ, δ and ε phases obtained by different processing methods. Among them, β phase PVDF presents outstanding electrical characteristics including piezo-, pyro-and ferroelectric properties. These electroactive properties are increasingly important in applications such as energy storage, spin valve devices, biomedicine, sensors and smart scaffolds. This article discusses the basic knowledge and character methods for PVDF fabrication and provides an overview of recent advances on the phase modification and recent applications of the β phase PVDF are reported. This study may provide an insight for the development and utilization for β phase PVDF nanofilms in future electronics.

## 1. Introduction

Poly(vinylidene fluoride), PVDF, with interesting scientific and technological properties is the typical representative of polymeric materials [1]. PVDF shows five crystalline polymorphs with α, β, γ, δ and ε phases depending on different processing methods [2,3]. Because of the antiparallel packing of the dipoles, the α and ε phases are non-polar [4]. The other three PVDF polymorphs are polar and show ferroelectric and piezoelectric properties. α, β and γ phases PVDF are mostly investigated for their properties and their extensive applications [5]. α phase PVDF is easy to obtain, which attracts wide interest to research melt crystallization of PVDF and α to β transformation [6]. Promoting β and γ phases in PVDF is an ongoing pursuit due to the most electrically active β and γ phases in the past studies [7,8]. Electroactive β and γ phases PVDF could be extensively utilized in spin valve devices, sensors, energy storage, etc. Moreover, the β phase PVDF was widely investigated for its interesting properties of the pyro-, piezo-, and ferroelectricity, and its inherent properties of light weight, mechanical flexibility, and easy processing. This paper mainly reviews the processing, properties and applications of the β phase PVDF.

## 2. Phases Characters of PVDF

**Figure 1:**
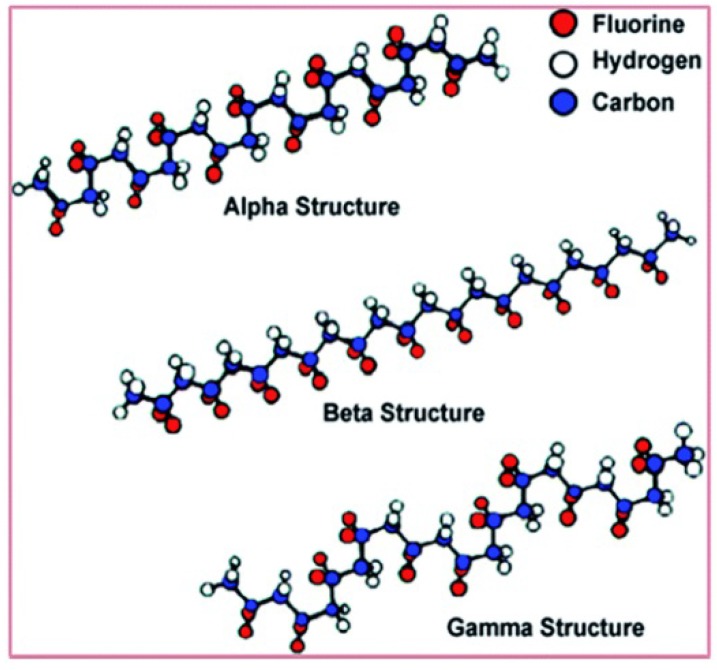
Diagrams of the chain conformation for α, β and γ crystalline phases of PVDF [13].

The chain conformation for different phases of PVDF, respectively α, β and γ phases is shown in Figure 1. The α phase is the one commonly obtained directly from the melt by crystallization. The all-trans planar zigzag conformation of β phase can induce a significant dipole moment. Furthermore, a large spontaneous polarization is generated by additive dipole moments. The piezoelectric effect of γ phase is weaker than β phase PVDF due to a gauche bond existed in every fourth repeat units [9]. So the β phase PVDF shows best piezo-, ferro- and pyroelectric properties among the five polymorphs of PVDF generally [10,11]. The lattice parameters of an orthorhombic β phase PVDF can be expressed as *a* = 8.47 Å, *b* = 4.90 Å, and *c* = 2.56 Å [12]. Three characterization methods, namely X-ray Diffraction (XRD), Fourier Transform Infrared Spectroscopy (FTIR), Differential Scanning Calorimetry (DSC) are used to study the formation and contribution of different crystalline phases of PVDF. 

**Figure 2:**
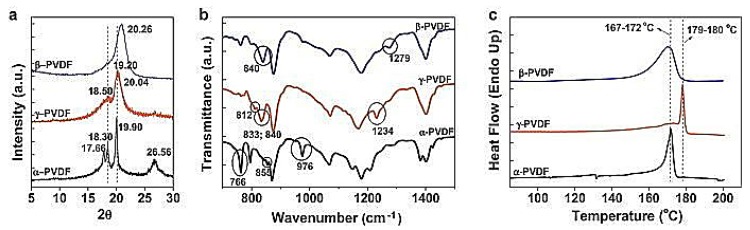
Synopsis of main experimental features of the different experimental techniques for correct identification of the PVDF phase: (**a**) XRD (K_α1_,γ = 1.5405600 Å); (**b**) FTIR; and (**c**) DSC [16].

The XRD and FTIR can implement the qualitative identification and quantitative analysis of the β phase (Figure 2a,b). DSC is a thermo-analytical technique that has been complementary to the other identification techniques to identify the crystalline phase of PVDF. The XRD spectra (Figure 2a) confirm that the peaks at 17.7°, 18.3° and 19.9° characteristic of the α phase of PVDF, and the peaks at 18.5°, 19.2°, 20.0°, characteristic of the γ phase. The peak at 2θ = 20.26° is related to the diffraction of β phase at (110) and (200) [14,15,16]. FTIR spectra of these three PVDF polymorphs have been investigated thoroughly and extensively [5,17,18,19]. Vibrational bands of α phase are at 530 cm^−1^, 615 cm^−1^ and 765 cm^−1^ and 795 cm^−1^, while vibrational bands exclusively of β phase are at 510 cm^−1^ and 840 cm^−1^, and the bands correspond to the γ phase are at 431 cm^−1^, 776 cm^−1^, 812 cm^−1^, 833 cm^−1^ and 1233 cm^−1^, as shown in (Figure 2b). The melting temperatures of the β crystallites and α phase PVDF are similar at 167–172 °C (Figure 2c), so DSC is used to calculate the crystalline percentage, but not to distinguish these three phases [15,20]. As a result, two of the above three measurement techniques are needed to identify a specific phase at least.

Though the IR spectrum of the α, β and γ phases includes many of the peaks due to overlapping absorptions, several unique peaks for different phases can be used for identification and quantification of the α, β and γ phases, such as their relative amounts. The IR absorption bands, characteristic of the α and β phases in each sample are at 766 cm^−1^ and 840 cm^−1^, respectively. The fraction of β phase, F(β), can be calculated using Equation (1) explained by Gregorio, and the formula is as follows:

(1) $$F\left(\beta \right)=\frac{X_{\beta }}{X_{\alpha }+X_{\beta }}=\frac{A_{\beta }}{\left(K_{\beta }/K_{\alpha }\right)A_{\alpha }+A_{\beta }}$$

where *X_α_* and *X_β_* in Equation (1) are representative for the mass fraction of α and β phases, absorption bands at 763 cm^−1^ and 840 cm^−1^ are represented by the *A_α_* and *A_β_* and the absorption coefficients are expressed by *K_α_* and *K_β_* at the particular wavenumber, respectively. This equation was used to quantify the FTIR results [5]. It is known that the peaks may be assigned to the different crystal phases. One issue is the overlapping of various peaks. The overlapping peaks could be deconvoluted using OMNIC software [21,22]. The aforementioned method can be used to calculate the crystalline phase content of PVDF when just two phases are present in the material (α and β, γ and β or α and γ).

## 3. Processing Techniques of β Phase PVDF

The β phase PVDF obtained by phase transitions, solvent casting, by the addition of nucleating fillers, or the development of PVDF copolymers have been researched extensively.

### 3.1. Phase Transition Methods

**Figure 3:**
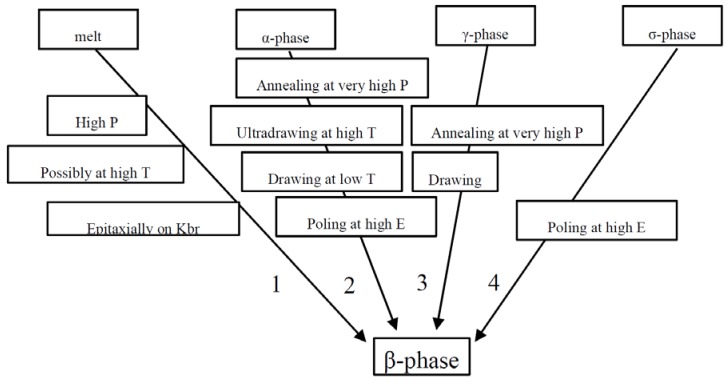
Four transition lines from different conformations to obtain β phase PVDF.

Phase transitions to obtain β phase PVDF can be divided into four methods depending on the processing conditions [23], as shown in Figure 3. 

The β phase obtained from melt needs high pressure, high temperature, epitaxial growth on Kbr or other specific conditions [23,24], as shown line 1 in Figure 3. The high-pressure-crystallized PVDF including both the low-melting and high-melting phase was indicated in a detailed study [25,26]. Quenching and then annealing of the PVDF films can induce the crystallization of β phase directly from the melt in the very early study of Yang et al. [26]. The α phase PVDF has been obtained directly from the melt [17,27,28,29,30]. The α to β phase transformation of PVDF(see line 2 in Figure 3) through the stretching annealing or poling process [31]. The fabrication of β phase PVDF by uniaxial drawing of α form film shows a typical piezoelectric character, which was proposed firstly by Kawai in 1969 [32]. This transformation idea has been widely investigated at different temperatures, stretching rate and tensile elongation [33]. The α to β phase conversion can be realized below 100 °C stretching temperatures, and about 3–5 stretch ratio in many studies [4,29,34,35], and the thickness of the prepared β phase PVDF films can range between several µm to hundreds μm. The piezoelectric properties of mechanically drawn PVDF are much improved by high-pressure crystallization PVDF film [36]. The maximum β phase (ca. 85%) could be achieved from α phase by Sharma et al. [37]. Strictly speaking, poling of PVDF films and fibers is done in order to obtain aligned β PVDF crystallites, and is in this sense not a method to induce the β phase. However, the β phase prepared by “drawing at low temperatures” is not piezoelectric due to the random orientation. The poling is needed to orient all β crystals into one direction to obtain a functional piezoelectric material [38]. Meanwhile, the transformation of α to β phase also occurs after poling with high electrical fields (100–400 mV/m) [39]. The γ phase is obtained near the melting point by annealing, or produced by casting method from dimethyl sulfoxide (DMSO), and δ phase can be obtained in strong electric fields by poling the α phase PVDF [40,41,42]. The γ to β phase transformation during uniaxial stretching is reasonable (see line 3 in Figure 3) because that of γ phase is a high energy intermediate between α and β phase (see line 3 in Figure 3). The γ phase can be transformed into β phase by poling at 120 °C, and the poled β PVDF films exhibit very strong and persistent piezoelectric effect up to 205 °C [36]. In addition, the δ phase can transform into β phase as shown line 4 in Figure 3 by poling at high electric field. The piezoelectric charge coefficient, *d*_33_, of −36 pm/V for the δ phase via solid-state-processing was reported very recently [43]. Interestingly, this value notably higher than electroformed δ PVDF (−15 pm/V) may open a new range of the piezoelectric PVDF materials [43].

### 3.2. Solvent Casting

A variety of experimental technique with specialized conditions were explored to obtain the β phase PVDF from different conformations (e.g., epitaxial growth on alkali halide substrates [24], mechanical stretching of the conventionally prepared films [35], electrical poling [31], crystallization at high pressure [26], etc.). However, only films of well-ordered several µm thicknesses can be prepared, and free-standing films with nanoscale thickness is very hard to fabricate using most of these methods. nanoscale thickness PVDF films can realize many microelectronics applications when deposited on a suitable substrate [44]. Thus, researchers have paid more and more attention to develop more convenient methods to obtain nanoscale films and nanofibers of β phase PVDF from solvent casting methods, such as electrospinning, Langmuir–Blodgett, spin-coating and solvent evaporation. Solvent-cast films of PVDF in dimethyl formamide (DMF), dimethylacetamide (DMAC), and DMSO, have been reported to bring about the formation of β phase PVDF [5,45].

#### 3.2.1. Electrospinning

Electrospinning technique has its superiority of versatility and consistency, much attention has been paid in the last decade to produce fibers in the submicron range using this method [46,47]. The β phase PVDF fibers can be formed from solution directly by electrospinning. This straightforward technique is relatively simple, fast, versatile and efficient. PVDF dissolved in DMF as electrospinning solutions has formed β phase PVDF nanofibers successfully in previous work [48,49,50]. The high ratio of stretching, which causes the α to β phase transition during the electrospinning is similar to the elongation during uniaxial mechanical stretching in some way. 

**Figure 4:**
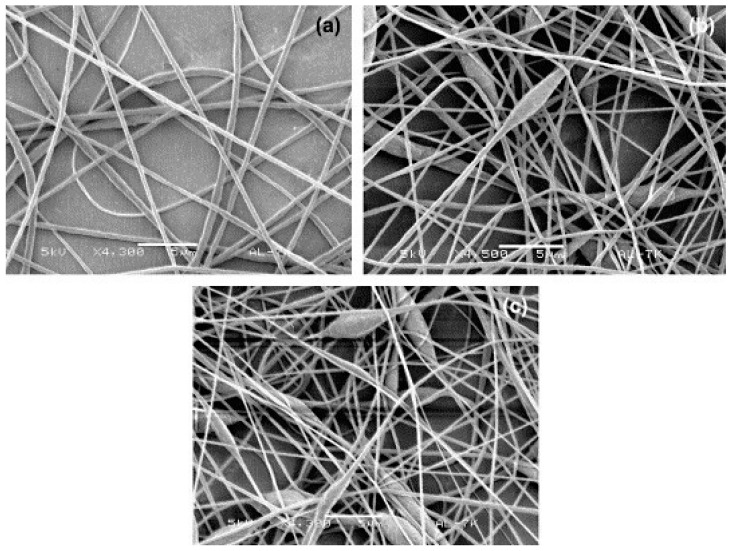
SEM micrographs of PVDF fibers obtained at 15 kV from 20 wt. % PVDF by electrospinning with different DMF/acetone weight ratios of: (**a**) 60/40; (**b**) 70/30; and (**c**) 80/20 [52].

PVDF fibers containing mainly β phase can be fabricated by control of the electrospinning conditional parameters such as electrospinning temperature, the solvent, the feeding rate, and distance between the tip and collector [51,52]. The fraction of β phase can reach 85% and the total crystalline fraction is higher than in the sample formed by the conventional methods we mentioned in the previous section in the work of Ribeiro et al. [46]. Figure 4 shows SEM micrographs of PVDF fibers directly produced from 20 wt. % PVDF solutions with different DMF/acetone ratios by electrospinning. The average fiber diameter was decreased with the increase of the DMF/acetone ratio and this phenomena can be interpreted to the higher boiling point and polarity of DMF than that of acetone [53]. PVDF fibers has attracted interest for various applications, including sensors, cell phones, batteries, biomedical applications, filtration, among others [54,55,56], and so many applications mean that the electrospinning technology for forming β phase PVDF fibers can achieve great development in the future.

#### 3.2.2. Langmuir–Blodgett

**Figure 5:**
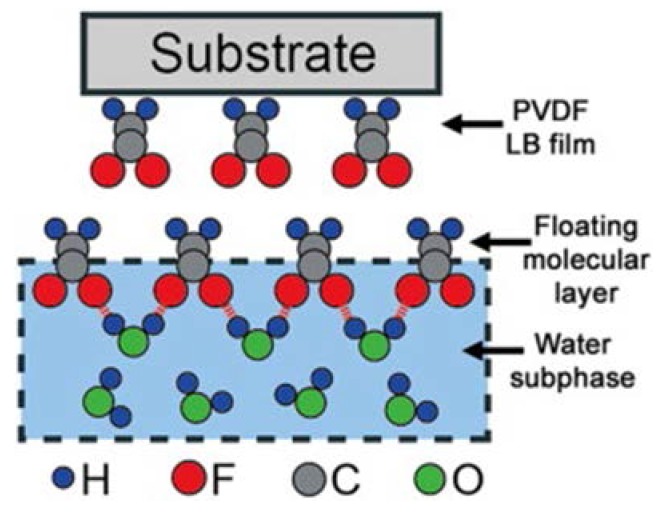
Schematic illustration of the oriented PVDF molecules and the subsequent transfer to form the LB film on a substrate [58].

Nanoscale films can be prepared by a monomolecular film assembly method, named the Langmuir–Blodgett (LB) technique [57]. Ferroelectric β phase PVDF is also formed directly during the LB deposition process. The forming of hydrogen bonds can result that the PVDF molecules on water surface are orientable with C–F groups, as illustrated in Figure 5, and a large value of effective piezoelectric coefficient of PVDF LB film, −49.4 pm/V, was acquired in this research [58]. A few nanometers thick β phase PVDF, is fabricated by LB technique at 40 mN/m surface pressure using ultra-pure water as the subphase, and poling process is not necessary [59].

**Figure 6:**
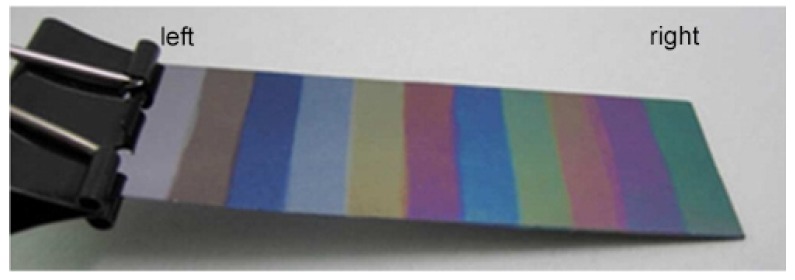
PVDF LB nanofilms with different layers, from 0 (**left**) to 200 (**right**) layers [61].

The sequential deposition of single monolayer enables the symmetry of the film to be precisely defined [60,61]. The regular layer structure and the smooth film surface can be proved by the beautiful interference colors of different layers of PVDF LB nanofilms, a related photograph is shown in Figure 6.

Zhu et al. [62] prepared highly oriented ferroelectric PVDF LB nanofilms by an assistance of amphiphilic nanosheets. PVDF films with thicknesses of several nanometers was prepared by LB technique [63], and the almost 100% β phase PVDF films could be fabricated and simultaneously the monolayer thickness can be controlled by a 2 nm through monolayer transfer [64]. 

#### 3.2.3. Spin-Coating

A thin, uniform polymer film can be produced from dilute solution on a planar substrate by a common method, known as spin-coating. Nanoscale films of β crystalline phase PVDF were directly obtained by heat-controlled spin coating [8,65,66]. Moreover, the spin-coating method is considered suitable to fabricate smooth polymeric coatings on an industrial scale for the microelectronics applications [8]. Benz et al. [45] prepared 2 μm thick PVDF films by spin coating PVDF solutions on single-crystal silicon wafers with acetone/DMF as a solvent. The main factors including humidity conditions and spin speed can determine the surface roughness of the thin films and the content of β phase. Cardoso et al. [67] demonstrate a simple and reproducible method to prepare electroactive β phase PVDF samples with the thickness controlled from 300 nm to 4.5 μm, and *d*_33_ from 0 to −21 pC/N with the electroactive phase content from 0 to 100%. 

**Figure 7:**
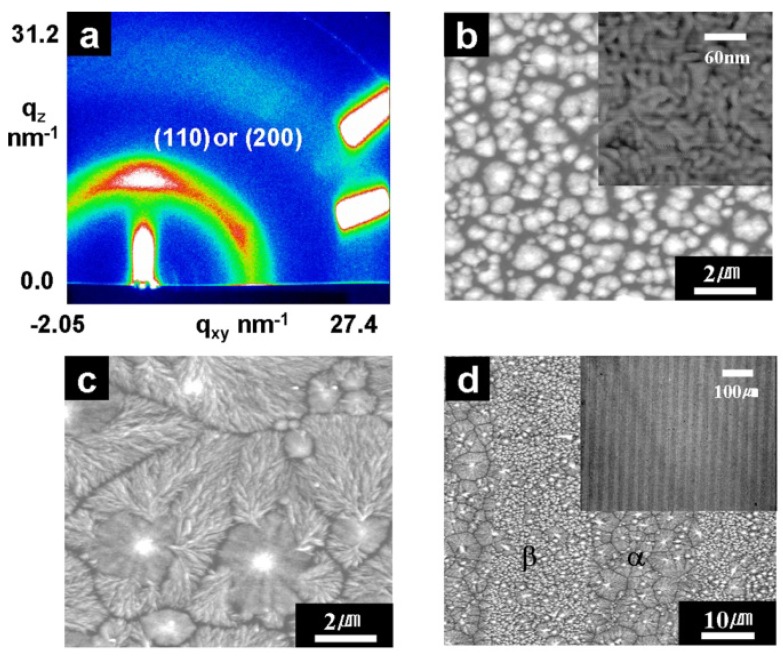
(**a**) 2D GIXD pattern and (**b**) AFM image of PVDF film with a 200 nm thick fabricated by spin coating on Au substrate and rapidly annealed at 150 °C; (**c**) AFM images of a PVDF film spin coating on treated Au substrate and rapidly annealed; (**d**) AFM image of αand βphase PVDF crystals [8].

In our work, 3 wt. % PVDF was dissolved in DMAc solution and coated on ITO substrates to prepare ferroelectric PVDF film successfully using a heat-controlled spin-coating setup [65]. The microstructure and micropattern of the PVDF film obtained by spin coating method can be found in Figure 7, and the ferroelectric phase of PVDF film fabricated by spin coating combined with treatment was maintained up to 160 °C [8]. The dense β phase dominant PVDF film can be obtained on silicon substrates by spin coating with addition of hydrate salt [68]. The apparent longitudinal *d*_33_ was −17.4 pm/V and the remnant polarization was 69.5 mC/m^2^. 

#### 3.2.4. Solvent Evaporation

The β phase thin films can be prepared by thermal evaporative deposition methods [69,70,71]. However, the thermal decomposition of the PVDF molecular chains seriously reduced the extent of ferroelectric response. Therefore, the solution deposition methods have been a possible use instead of the evaporative deposition [72,73,74,75]. Many reports have proved that the solution processing can produce devices based on silicon at significantly lower cost than conventional methods. The β phase obtained by solution trends to present a high porosity and an opaque appearance leads to a decrease of the electrical and mechanical properties. The pores in original sample can be eliminated after applying pressure perpendicular to the surface of β phase film at 60 °C at elevated temperature [74]. Oriented β phase films were obtained by drawing exclusively β phase films with subsequent pressing at 60 °C from DMF solution [75].

### 3.3. Copolymers

A wide variety of copolymers have been synthesized by incorporation PVDF with the trifluoro ethylene (TrFE), hexafluoropropylene (HEP) comonomer, chlorotrifluoroethylene (CTFE) or other polymers. The β crystalline phase of PVDF has been detected in the poly(tert-butyl methacrylate)-block-PVDF-block-poly(tert-butyl methacrylate) triblock copolymers [76]. The change from α crystalline phase to the β crystalline phase was also confirmed by FTIR and XRD measurements in the block copolymers [77]. The development of poly(vinylidene fluoride-trifluoro ethylene) P(VDF-TrFE) has verified that the material obtained can possess the electroactive property. This copolymer also shows the Curie Temperature (*T_c_*) lower than the melting temperature (*T_m_*), and, in this way, allows the study of ferroelectric (FE) to paraelectric (PE) phase transition [78,79].

Ohigashi et al. [80] prepared “single crystalline films” of ferroelectric P(VDF-TrFE) (75/25 molar ratio) with the 5–100 μm thickness successfully in 1995. Poly(vinylidene fluoride-co-hexafluoro propene), P(VDF-HFP) has been mainly studied for applications on the area of polymer electrolytes, such as using in rechargeable lithium batteries and for the production of membranes for organophilic pervaporation [81,82]. PVDF has also been modified by the introduction of chloride trifluoride ethylene (CTFE) on the polymer chain, producing the P(VDF-CTFE) copolymer. The bulky CTFE can induce the structure of copolymer loose, which can make the orientation of dipoles easier under external electric field. The piezoelectric constant, *d*_33_ for this copolymer can reach the value of 140 pC/N [83,84].

### 3.4. Composites: Inclusion of Fillers within the Polymer Matrix

The β phase PVDF has been obtained by the addition of BaTiO_3_ [85,86], clay [87], hydrated ionic salt [45], PMMA [88], TiO_2_ [89], CoFe_2_O_4_ [90] or nanoparticles such as palladium, ferrite or gold [91] as nucleating fillers. It is also reported that β phase PVDF nanocomposites can be fabricated with the assistantance of ionic liquid [92,93]. 

The direct nucleation of the β phase PVDF can save processing some steps if the material obtained by extrusion processes and micro technology compatible technologies in the desired size and shape. The presence of BaTiO_3_ can make β PVDF phase nucleate, and the nucleation is dependent on filler size strongly [86]. Martins et al. [94] prepared PVDF/ferrite nanocomposite by addition NiFe_2_O_4_ of and CoFe_2_O_4_ nanoparticles to nucleate electroactive β phase PVDF for multiferroic and magnetoelectric applications, and 50 wt. % of NiFe_2_O_4_ and 5 wt. % of CoFe_2_O_4_ can guarantee to obtain more than 90% β crystalline phase. They found the content of β phase increases with the increasing concentration of CoFe_2_O_4_ and NiFe_2_O_4_ in a different way in the following year [95]. The negative electrostatic charge of nanoparticles is used to elucidate the nucleation of β phase. The CH_2_ bonds of the PVDF and the negatively charged ferrites surface have a strong interaction, which induces an extended TTTT conformation aligned on the surface of nanoparticles and results in crystallographic phase formation of the β phase PVDF. 

## 4. The Electroactive Properties of the β Phase PVDF

The piezoelectric property of PVDF was first found by Kawai in 1969 [32], and the pyroelectric property of PVDF was found by Bergman et al. [96] and by Nakamura and Wada [97] two years later. PVDF has been investigated widely because of its pyro-, piezo- and ferroelectric effect within the last decades [98]. 

### 4.1. Piezoelectric Effect

**Figure 8:**
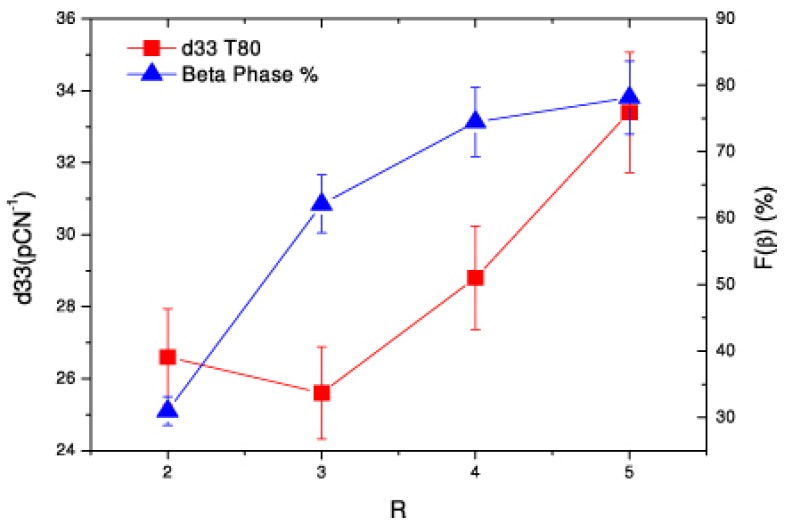
*d*_33_ and F(β) (%) for samples depending on stretching ratios R at 80 °C [100].

A Mach–Zehnder interferometer was used to measure the sample strain for the measurement of piezoelectric property [99]. The piezoelectric coefficient *d*_33_ can be defined by Equation (2), and the strain was induced by the applied electric field.

(2) $$d_{33}^{T}={\left(\frac{\partial S_{3}}{\partial E_{3}}\right)}_{T}$$

where the strain *S* is caused by the external electric field *E*, and *T* is the constant stress. The direction of spontaneous polarization is along the *x*_3_ axis. The three-dimensional tensor *d_ijk_* is represented by two-dimensional *d_il_*, where *l* represents the six different combinations of the indices (*j*, *k*). Figure 8 shows the *d*_33_ values increasing with the β phase content of samples, and both of them increase with increasing stretching ratios at 80 °C [100].

The decreasing *d*_33_ of PVDF indicated that thermal depoling can be caused by annealing at an elevated temperature. The polymer retains 32% of its original *d*_33_ value after annealing at 140 °C. The decrease of *d*_33_ may also orginate from the random orientation of β phase PVDF. So thermal poling could orient all beta-crystals into the same direction [101]. (CH_2_-CF_2_)*_n_* is the chemical structure of PVDF molecules, and after poling the CF_2_ dipoles are aligned normal to the surface of the film and form a residual polarization. The *C*_2*v*_ (2 mm) represents the symmetry of poled films, if the *z* axis is assigned normal to the surface of films in the poling direction, the *x* axis along with the direction of elongation, and perpendicular to the *x* and *z* axes can confirm the *y* axis. Five piezoelectric coefficient observed values are *d*_31_ = 20 pCN^−1^, *d*_32_ = 1.5 pCN^−1^, *d*_33_ = −32 pCN^−1^, *d*_15_ = −27 pCN^−1^, and *d*_24_ = −23 pCN^−1^, are finite, respectively [102]. 

### 4.2. Pyroelectric Effect

**Figure 9:**
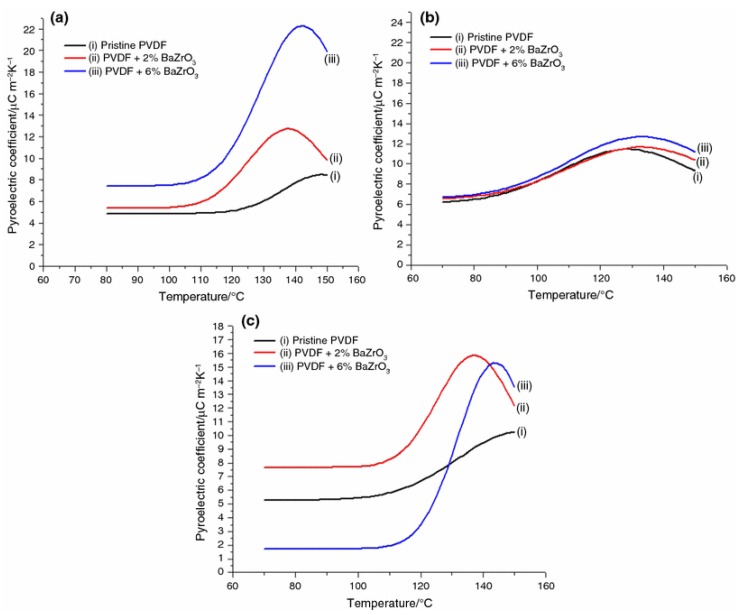
Pyroelectric coefficient of PVDF and PVDF nanocomposites depended on temperature of (**a**) 1 °C/min, (**b**) 2 °C/min, and (**c**) 3 °C/min heating rate [108].

Pyroelectric effect is defined as the spontaneous polarization depended on the temperature of certain anisotropic solids [103]. In principle, there are two ways that the pyroelectric coefficient can be measured: either by holding the size and shape of the sample constant while the temperature is changed or by allowing the sample to expand and change shape freely as a result of thermal expansion. In the second experiment there is an secondary pyroelectricity resulting from piezoelectricity [104]. Pyroelectric measurements can be implemented by using both slow heating/cooling method and Chynoweth method [105,106]. The different thermal expansion coefficient between the substrate and the polymer makes the film expand and contract just perpendicular to the surface, so two contributions are included in the measured pyroelectric coefficient *p*_3_(*eff*) in Equation (3), namely the primary and the secondary effect: [107]

(3) $$p_{3}\left(eff\right)={\left(\frac{\partial P_{S}}{\partial T}\right)}_{S}+\frac{d_{33}^{T}\alpha_{3}^{S}}{S_{33}^{S}}=\left(\frac{I}{A}\right){\left(\frac{\partial T}{\partial t}\right)}^{-1}$$

where $p_{3}\left(eff\right)$ is the pyroelectric coefficient, *S* is the strain, *T* is temperature, $d_{33}^{T}$ is the stress-free piezoelectric coefficient, $\alpha_{3}^{S}$ is the thermal expansion coefficient, $S_{33}^{S}$ is the elastic compliance coefficient, *A* is the surface area, the pyroelectric current is represented by *I*, and the rate of temperature change is expressed by *∂T/∂t*. The temperature was controlled by a microprocessor [99]. Figure 9 shows that the pyroelectric coefficients (*P*) are depended on the heating rate, concentration of BaZrO_3_, and temperature [108].

### 4.3. Ferroelectric Effect

**Figure 10:**
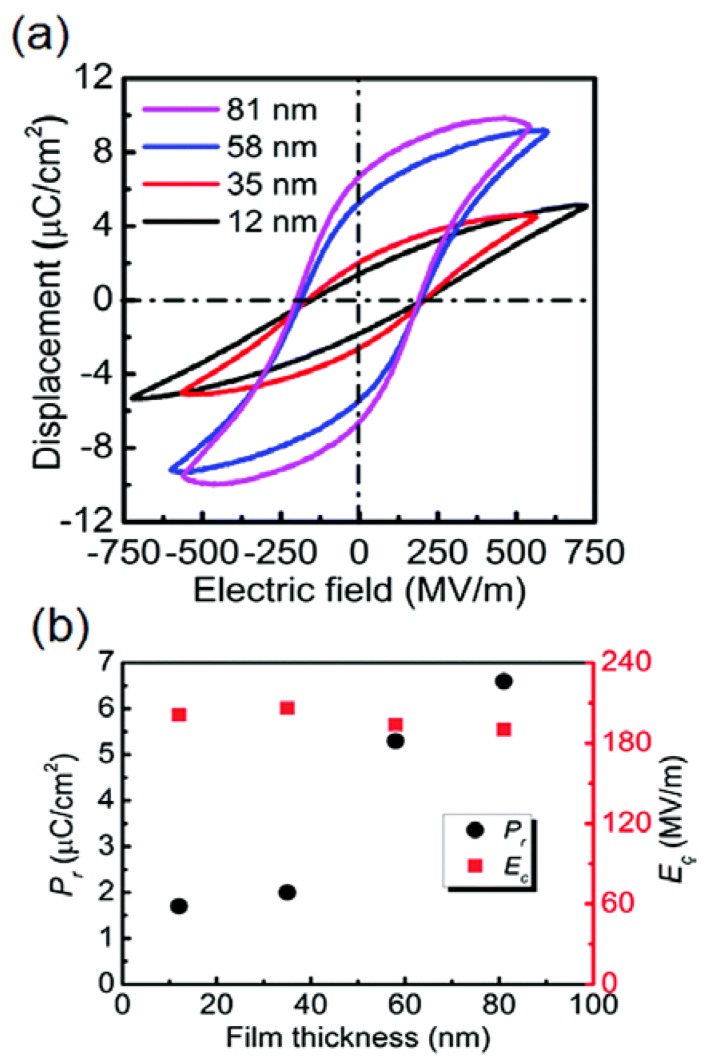
(**a**) The D-E Hysteresis loops and (**b**) coercive field (*E_c_*) and remanent polarization (*P_r_*) of PVDF nanofilms dependent of different film thicknesses [110].

The ferroelectric property of PVDF was anticipated since the large piezoelectric effect was reported for PVDF. In 1974, the observation of the hysteresis loop was successfully performed [80]. PVDF has extremely potential to be used in multi-functional MR devices as an organic ferroelectric material. A previous study has shown that the D-E hysteresis loop of PVDF is independent on the frequency of the applied field between 10^−2^ and 10^−4^ Hz at room temperature [109]. The D-E hysteresis loops are shown in Figure 10a, and the remanent polarization (*P_r_*) and coercive field (*E_c_*) dependent of the different film thicknesses of PVDF LB nanofilms are shown in Figure 10b. A *P_r_* of 6.6 μC/cm^2^ for PVDF nanofilm with 81 nm thickness was obtained without post-treatment, and ferroelectricity was detected first in a PVDF nanofilm with 12 nm thickness, indicating a potential application of PVDF films at low-voltage [110].

The ferroelectric effect of PVDF originates from the dipole moments in the PVDF molecule. The dipole moments stem primarily from the presence of fluorine atoms which have the strongly electronegative [111,112]. The PVDF thin films fabricated from the melt or a solution have no ferroelectric effect because the dipole moments cancel in the crystal structure and stereo-chemical conformation, additional steps such as stretching, using P(VDF-TrFE) instead of PVDF are needed to make these films ferroelectric [113]. Ferroelectricity has many features similar to ferromagnetism including a shift of the phase transition temperature *T_c_*(*E*) with applied electric field and the disappearance of the spontaneous polarization above the zero-field Curie point *T_c_*_0_ [107]. In this following report, we will mainly discuss the applications of β phase PVDF. Other studies about PVDF with various phases can be found in the literatures [114,115,116,117,118,119,120,121,122,123,124,125,126,127]. These investigations would also be very useful for future applications.

## 5. Applications of the β Phase PVDF

Kawai found the tensile piezoelectricity of PVDF in stretched and poled films in 1969 [32]. In addition, then PVDF have many applications as ultrasonic transducers, batteries, actuators, chemical warfare protection, magnetoelectric, filters, and in the biological fields.

In 1972, ultrasonic transducers making use of the piezoelectric constant *d*_33_ of PVDF, were first produced. Electroacoustic transducers utilizing the piezoelectric constant *d*_31_ of PVDF, were marketed in 1975 [128]. The cell response and fibronectin adsorption on electroactive PVDF films make PVDF possible to be active substrates [129]. Various applications of PVDF and its copolymers have been greatly developed during almost five decades. Here we chose several promising applications which were widely concerned by researchers recently to introduce.

### 5.1. Sensor and Actuator Applications

Actuators have potential applications and attracted much attention in recent years because the sensor can convert a mechanical variable into a measurable electrical quantity. PVDF has been quite extensively applied in sensing and actuator due to its inherent piezoelectric effect. The applications of Actuator based on PVDF can be found in acoustic emission monitoring, controlled displays, robots and artificial muscles [130,131,132,133], and a series of studies have been done to explore the use of PVDF such as weight, pressure sensor [134,135,136,137,138,139]. Active vibration control by a laminated PVDF actuator is studied [132], and an SGO/PVDF bilayer actuator can respond to moisture, light and heat with excellent stability and repeatability [140]. The PVDF/graphene actuators show faster response time and larger bending deformation than the pure PVDF actuator [141]. Fully plastic actuator through layer-by-layer casting with ionic-liquid-based bucky gel was reported by Takanori et al. [142]. They also proposed a new two-step process for preparing a gelatinous mixture composed of the SWNTs. In this study, the ionic liquids and PVDF-HFP as a polymer support improved the performance of the actuator [143].

**Figure 11:**
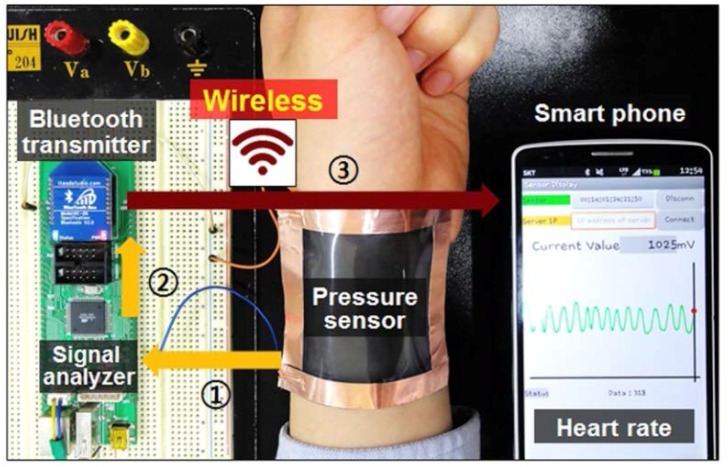
Photograph of wearable and wireless pressure sensor for heart rate monitoring [147].

A PVDF film microforce sensor was designed and fabricated to measure the strength of fiber bonds successfully, and this microforce sensor is able to measure the forces up to 10 mN [144]. Choi et al. [145] use macro fiber composite actuators and PVDF sensors to enhance the damping performance of the rotating composite thin-walled beam. A quartz crystal microbalance modified by PVDF was used to detect the dimethyl methyl phosphonate (DMMP) vapor [146]. Highly sensitive, wearable and wireless pressure sensor successfully fabricated based on the zinc oxide (ZnO) nanoneedle/PVDF hybrid film was shown in Figure 11. 

**Figure 12:**
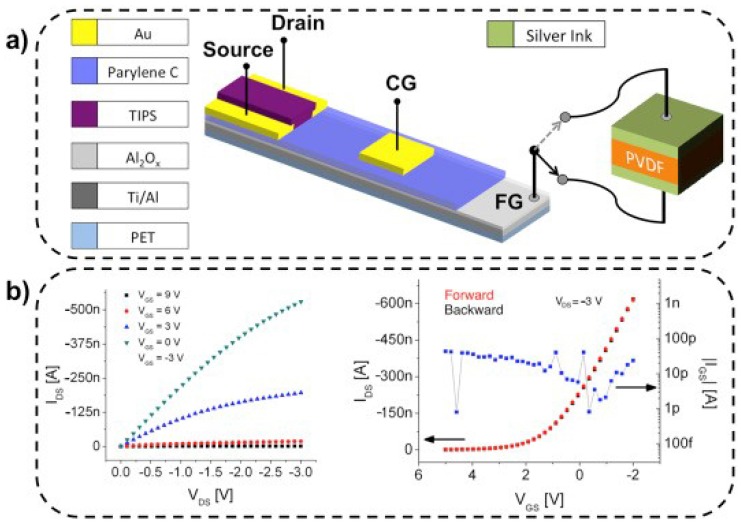
(**a**) The designed structure of the highly sensitive tactile sensor and (**b**) Output and input characteristics of designed device for pressure sensing [152].

Kim et al. make a significant contribution to fabricate sensors based on PVDF fabrics [148]. Several factors should be taken into account in fabrication of the force sensors, such as electrospinning parameters, fiber alignments, and materials of the electrodes [149]. The addition of nanofillers can increase the β phase PVDF commonly in composites, and in turn can improve the piezoelectric response for sensor and actuator applications [150,151]. A highly sensitive tactile sensor based on an Organic Charge Modulated FET (OCMFET) and PVDF has been reported by Spanu et al. [152]. The structure of the sensor can be found in Figure 12a, and the output and input characteristics for pressure sensing was shown in Figure 12b. This proposed device can transduce applied pressure as low as 300 Pa range with good reliability in a wide frequency, opening the possibility to fabricate tactile transducers with highly sensitive using large area fabrication techniques.

### 5.2. Spin Valve Devices

Various organic ferroelectric materials have existed, but much attention is focused on PVDF and its copolymer due to its several advantageous properties including short switching time, relatively large remnant polarization, and good thermal stability [113]. 

**Figure 13:**
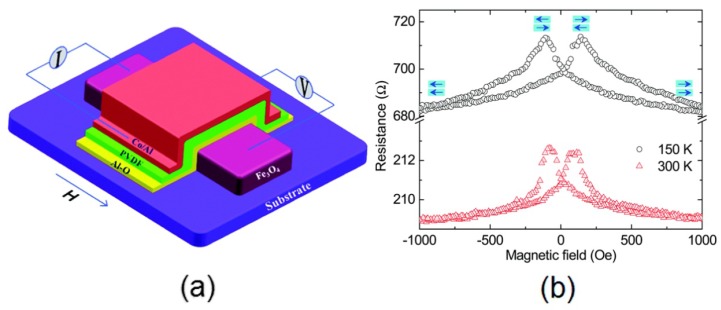
(**a**) Schematic diagram of the spin valve device with an Fe_3_O_4_/AlO/PVDF/Co./Al stacking structure; and (**b**) Magnetoresistance curves for the device at 300 K (red triangles) and 150 K (black circles) [64].

Many efforts are paid to fabricate nonvolatile and rewritable memory devices using organic PVDF. Memory devices utilize the hysteresis by associating the polarization states to form the basis for most logic circuits. Some of these efforts are focused on metal/organic semiconductor/metal junctions [153], field-effect transistors [154], and switches [155]. PVDF and P(VDF-TrFE) can be placed between the capacitor electrodes as the ferroelectric medium [111]. Ferroelectric PVDF switching has an activated process depending on not only the temperature, but the applied field strength. La_0.6_Sr_0.4_MnO_3_/PVDF/Co.-based multiferroic tunnel junctions shows a tunable spin polarization at the PVDF/Co. interface depending on the ferroelectric polarization of PVDF. Remarkably, a change of tunneling magneto-resistance sign found when the PVDF polarization is reversed can be explained by the ferroelectric change of spin-polarization in the second PVDF layer close to PVDF/Co. interface, which acts as an efficient spin-filter for electron tunneling through PVDF barrier [156]. Ferroelectric PVDF have been used to fabricate magnetoresistance (MR) devices as spacer layers at low temperatures. The β phase PVDF films were fabricated by the LB technique and then used to prepare the organic spin valve devices successfully (Figure 13a) by Zhang et al. [64]. It is valuable using PVDF for multi-configuration spin control to fabricate future flexible spin devices, more than that, they have achieved an MR ratio over 2.5% at room temperature for the PVDF spin devices (Figure 13b).

Furthermore, Zhu et al. [62] report a PVDF ferroelectric nanocapacitor which shows asymmetric hysteresis loops, and it results in different remnant polarization values of −15 μC/cm^2^ for −*P_r_* and 6.0 μC/cm^2^ for +*P_r_*. This peculiar discovery opens up the probable applications of ferroelectric PVDF for the diode.

### 5.3. Magnetoelectric Materials

**Figure 14:**
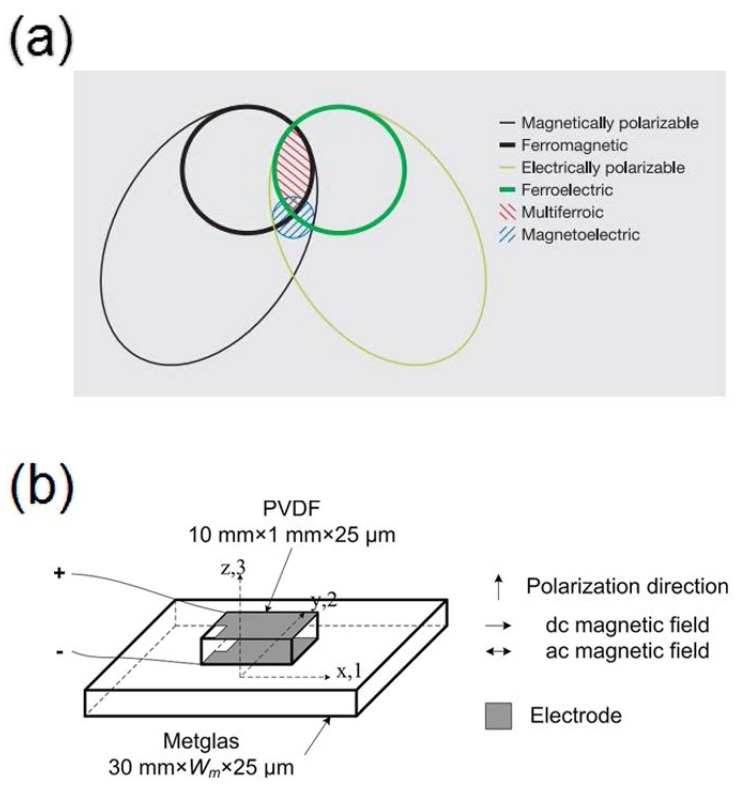
(**a**) The relationship between multiferroic and magnetoelectric materials [157] and (**b**) Diagrammatic sketch of Metglas/PVDF composite laminates [170].

The induced magnetization by an applied electric field variation as well as the electrical polarization of a material in the presence of an applied magnetic field can be defined as magnetoelectric (ME) effect [157,158,159]. The magnetoelectric (ME) effect is the bridge between the electric properties and magnetic properties of materials. A strong ME effect at room temperature is generally obtained by combining piezoelectric and magnetostrictive components [160]. ME materials using piezoelectric polymers appear to be very suitable due to their versatility of production, flexibility, and low cost [161]. The magnetoelectric materials can be distinguished from other materials in Figure 14a. Polymer-based ME materials are an innovative, challenging and interesting research field, which should bridge the gap between fundamental research and practical applications. Laminated composites, nanocomposites and polymer as a binder composites are the three types of polymer-based ME materials [159]. The ME materials have immensely potential applications in numerous areas such as spintronics, multiple-state memories, actuators, transformers, information storage, sensors, diodes, among others [162,163,164,165,166]. The bilayer PVDF/Metglas laminates magnetic sensors for detection of weak dc magnetic field shows quite high sensitivity [160]. The ME laminates consisting of Metglas/PVDF unimorph has been studied and shows an excellent sensitivity to small variations in both ac and dc magnetic fields [167]. Furthermore, the energy-harvesting applications using polymer-based ME composites have recently been proposed by harvesting energy from wind, sun, electromagnetism, etc. [159,168]. An energy-harvesting device was designed using PVDF as the piezoelectric element and Fe_64_Co_17_Si_7_B_12_ as amorphous magnetostrictive ribbons [169]. 

Intensive works and more attention are devoted to ME material after the first report on Metglas/PVDF laminate nanocomposites [167]. Schematic diagram of Metglas/PVDF composite laminates was showed in Figure 14b. The sheet aspect ratio values of α_31_ = 21.46 V cm^−1^ Oe^−1^ were obtained in the PVDF/Metglas laminate at 3 Oe DC magnetic field and a frequency of 20 Hz [170]. A flexible planar structure of amorphous ferromagnetic Metglas—piezopolymer PVDF, and the ME coefficient was estimated as α_E_ = *u*/(*bh*) ≈ 0.47 V/(cm·Oe) at optimum bias field Hm [171]. The composite laminates of PVDF and the magnetostrictive layer have the strong coupling effect, which can induce large ME coupling coefficients at room temperature and have attract much attention of researchers [159,167,170,171,172,173]. 

### 5.4. Energy Harvesting Applications

**Figure 15:**
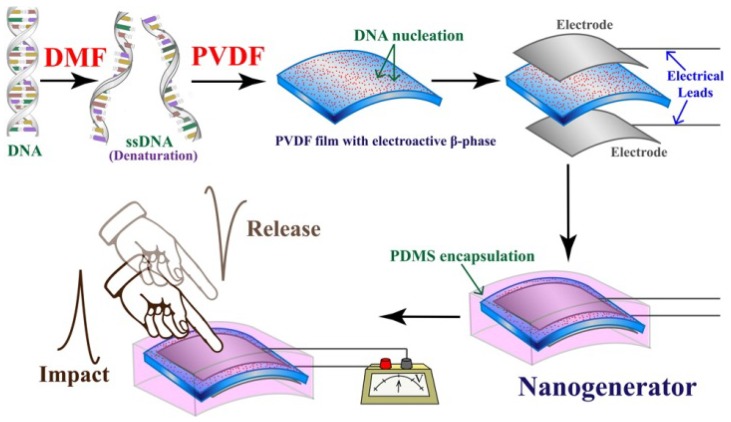
The structure of a flexible nanogenerator for Portable Electronic Devices [178].

Energy harvesting applications of piezoelectric and pyroelectric PVDF are developed rapidly which concentrate on devices, including ‘nanogenerators’ [174], nanoscale materials and various potential devices. This includes the conversion of mechanical vibrations to electrical energy via piezoelectric effect and the convert thermal fluctuations into electrical energy by pyroelectric effect [13,175,176,177]. The hybrid systems for harvest energy from multiple sources is possible since piezoelectric effect and pyroelectric effect are always present simultaneously. Figure 15 shows a flexible nanogenerator (NG) fabricated with a PVDF film [178]. Flexible piezoelectric nanogenerators were fabricated based on BaTi_(1−*x*)_Zr*_x_*O_3_ and a PVDF polymer matrix using an ultrasonication probe process [179].

A novel energy harvesting backpack using PVDF was developed that can generate electrical energy from the differential forces between the backpack and the wearer by Farinholt et al. [180]. An energy harvesting comparison of PVDF and ionic polymer transducer was proposed [181]. Sun et al. [182] reported that the PVDF microbelts can offer sufficient electrical energy for small electronic devices. Li et al. [183] reported a Bi-resonant structure with PVDF film for piezoelectric energy harvesting, which can improve the conversion efficiency from vibration to electricity by 40–81% significantly. The piezoelectric coefficients of its copolymers are larger than PVDF such as PVDF-TrFE (*d*_31_ ≅ 25 pC/N and *d*_33_ ≅ −30 to −40 pC/N) [184]. So many copolymers have been previously investigated for energy harvesting application. A flexible piezoelectric nanogenerator based on PVDF-TrFE thin film was fabricated, and it can exhibit reasonable electrical outputs and good stability [185]. Lallart et al. [186] evaluated the energy scavenging abilities using electrostrictive polymer P(VDF-TrFE-CFE), and they extended their work to make the practical application more realistic by researching the ac-dc conversion for energy harvesting [187]. 

### 5.5. Tissue Engineering

**Figure 16:**
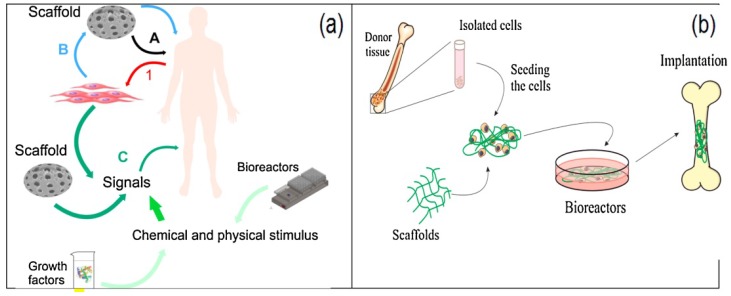
(**a**) Schematic diagram of the different strategies in the tissue engineering field: 1—the cells harvested directly from the patient; A—scaffold implanted directly; B—cells cultured in scaffold and then implanted; C—cells cultured in scaffold with appropriate signal and then implanted. (**b**) Tissue engineering strategies for bone regeneration [192].

The piezoelectric properties of living tissue have been also reported and PVDF as a piezoelectric material has attracted more attention in tissue engineering research [188]. Piezoelectricity can be found in different parts of the human body, such as bone, ligaments, etc. [189,190,191,192,193]. Tissue engineering has emerged for tissue repair and regeneration as an alternative method, as presented in Figure 16a. Figure 16b shows one promising strategy to repair or regenerate damaged bone. 

Young et al. fabricated microporous PVDF membranes for nerve tissue engineering by immobilizing L-lysine covalently on the surface of PVDF membrane [194]. The collagen from non-mammalian sources could be used to confer bioactivity to PVDF with hemocompatibility to bovine collagen and comparable cell-material interactions [195]. Therefore, the tissue engineering strategies based on PVDF materials show potential applications for human health.

### 5.6. Modelling Studies for PVDF

Modelling work for PVDF has been performed and the results are very interesting [196,197,198,199]. The atomistic modelling can be used to understand the changes at a molecular level on account of the poling process [39]. Holman and Kavarnos reported that the increase of both the trans bond number and structural order in P(VDF-TrFE) during the cooling process from the melt [200]. Bóhlen and coworkers have proven copolymerization of VDF with TrFE can form the all-trans PVDF conformations [197]. Moreover, the adhesion of single wall carbon nanotubes and the PVDF can be studied by molecular dynamics calculations [201]. In addition, They also proposed that the combination of atomistic simulations and macro-scale experiments could study the torsion angle distribution of PVDF in the melt [202]. The first-principles methods were also used to investigate the PVDF spin devices [203,204]. Their results indicate that β phase PVDF has enormous potential in organic spintronics for nonvolatile data storage and low-power electronics.

### 5.7. Reversible 3D Shape Transformation of the PVDF Film

The materials capable to translate the environmental stimuli into desirable shape changes have a bright prospects in emerging fields such as soft robotics, sensors, energy harvesting, multifunctional bio-scaffolds and metamaterials [205,206]. Responsive shape changes of a porous polymer film driven by gradients of electrostatic complexation have been reported recently [207]. Responsive shape changes of a carbon nitride polymer film actuated by anisotropic crystalline orders was reported more recently [208].

**Figure 17:**
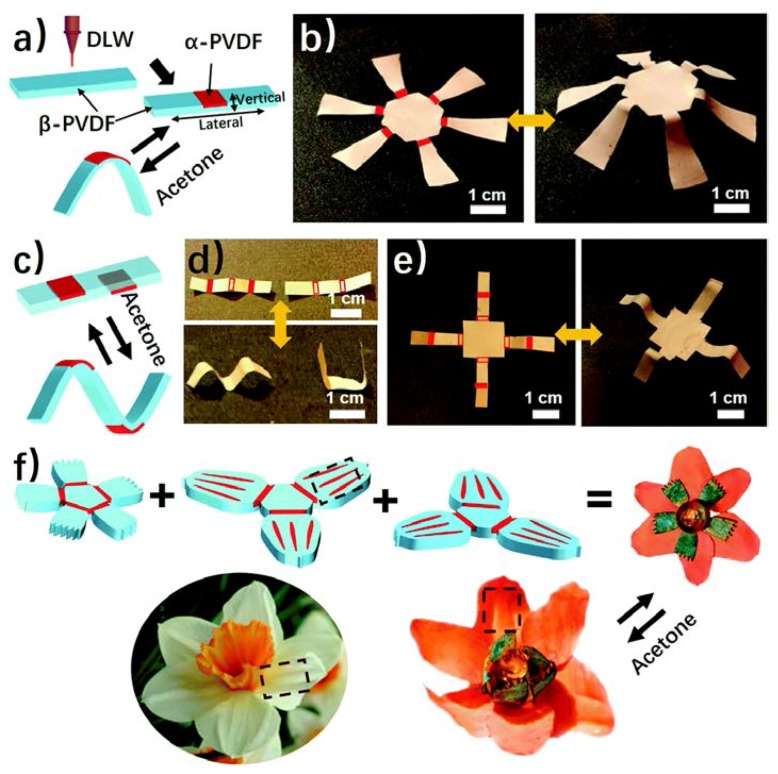
(**a**) Scheme of using direct laser writing to pattern the α phase domains in β phase PVDF film. (**b**) A photograph of the 3D structure transformed from a laser-patterned origami film. (**c**) A scheme of bidirectional folding of β phase PVDF film, resulting inform laser patterning on both sides. (**d**) A “MU” logo and (**e**) A 3D structure transformed from a to β phase PVDF film with laser patterning on both sides. (**f**) A PVDF blooming flower evolving from assembled multilayered PVDF films with each layer having origami patterns consisting of α and β phase origami patterns [209].

Very recently Deng et al. [209] report a new responsive film which possesses an anisotropic spatial distribution of the α and β phase PVDF, and this PVDF film can response to organic vapors in high sensitivity and a fast response rate. The crystal phase anisotropy can be programmed easily in different directions and locations of the PVDF films by a direct laser writing technique (Figure 17a). Experimental and simulation results show the shape change is attributed to the distinct absorption abilities for acetone between α and β phase PVDF. Many complex structures can be obtained by different folding creases on the PVDF films, including a complex shape transformation from 2D to 3D (Figure 17b–f). This responsive shape change will inspire researchers to explore more polymeric materials and further investigate for the underlying applications in artificial muscles, soft robotics sensors, etc.

## 6. Conclusions and Perspective

The PVDF is one of the most challenging and interesting materials for the development of advanced applications due to its high dielectric constant, piezo-, pyro- and ferroelectric effects. Recent advances have been presented for β phase PVDF with the recent application possibilities in areas such as magnetoelectric materials, sensors, filtration membranes and biomedical applications. Despite significant progress has been made, some critically technical points are still needed for consideration for further applications. In general, they mainly include the following items: (i) The effective modification of homogeneous surface of PVDF films. A large roughness is harmful for electron transfer in electronic devices, which leads to a leak of current and reduce the device lifetime. (ii) The coupling mechanism for PVDF/electrodes interface should be further studied because the ferroelectric nature of PVDF likely induces the new barriers by gathering electron charge. This is important for the development of organic spin devices operated at room temperature. (iii) Seeking new techniques to enhance the efficiency for PVDF applications in Energy harvesting, tissue engineering, etc. This would be key step for the actual applications of PVDF devices. (iv) In addition, the geometries of PVDF with different crystal phases are needed to be studied in detail. This is very useful to get an insight on the relations between the morphology and the physical properties.
